# 4-octyl itaconate alleviates LPS-induced inflammation in Sertoli cells by inhibiting excessive autophagy

**DOI:** 10.1016/j.redox.2026.104307

**Published:** 2026-07-17

**Authors:** Yuan Li, Xianglong Wang, Haijuan Yang, Feng Jiang, Meihua Wang, Yue Wang, Dong Niu, Huaming Xi

**Affiliations:** College of Animal Science and Technology & College of Veterinary Medicine of Zhejiang A&F University, Hangzhou, 311300, China

**Keywords:** 4-octyl itaconate, Sertoli cell, Inflammation, Autophagy, ULK1

## Abstract

Bacterial orchitis is a major cause of male infertility, yet effective therapies remain limited. Although the itaconate derivative 4-octyl itaconate (4-OI) possesses potent anti-inflammatory properties, its role in testicular inflammation is unclear. Here, we investigated the protective effects and mechanisms of 4-OI in lipopolysaccharide (LPS)-induced inflammatory injury using Sertoli cells and a mouse model of acute orchitis. LPS activated NF-κB/NLRP3 signaling and induced excessive autophagy in Sertoli cells, resulting in oxidative stress, apoptosis, disruption of tight junctions, and functional impairment. 4-OI markedly suppressed NF-κB phosphorylation and NLRP3 activation, reduced mitochondrial oxidative stress, and improved cell viability. Mechanistically, 4-OI inhibited excessive autophagy by downregulating UNC-51-like kinase 1 (ULK1) and autophagy-related proteins, thereby limiting autophagic flux. Consequently, Sertoli cell functional markers, tight junction integrity, and mitochondrial homeostasis were restored. *In vivo*, 4-OI alleviated testicular histopathological damage, reduced germ cell apoptosis, improved sperm quality, preserved blood-testis barrier integrity, and enhanced spermatogenic activity. Collectively, these findings identify ULK1-associated excessive autophagy as a key mechanism of inflammatory testicular injury and demonstrate that 4-OI protects against orchitis-induced reproductive dysfunction, highlighting its therapeutic potential for inflammation-associated male infertility.

## Highlights

• 4-OI effectively alleviates inflammation by inhibiting NLRP3 inflammasome activation.
• 4-OI inhibits NLRP3 inflammasome activation by reducing autophagy.
• 4-OI-inhibited excessive autophagy maintained the tight junctions in Sertoli cells.

## Graphical abstract

**Figure:**
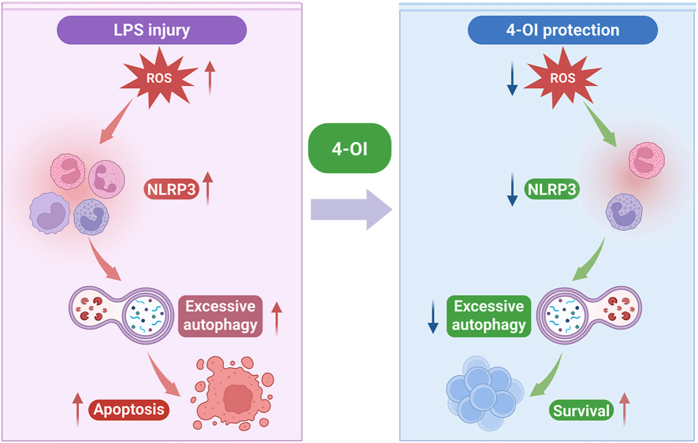

## 1 Introduction

The testis, as the core organ of the male reproductive system, is crucial for spermatogenesis due to the homeostasis of the immune microenvironment. The testis maintains an immune-privileged state through the physical isolation of the blood-testis barrier (BTB), the secretion of immunosuppressive factors (such as TGF-β1 and IL-10), and the infiltration of immune cells to protect germ cells from autoimmune attacks [1,2]. However, when the body is infected by pathogenic microorganisms or physical and chemical damage, the immune privilege balance is broken, triggering a testicular inflammatory response [[3], [4], [5]]. Numerous studies have demonstrated that pathogenic microorganisms can induce orchitis and ultimately impair male fertility [6]. Moreover, increasing evidence indicates that environmental toxicants, endocrine-disrupting factors, and aging also contribute to male reproductive dysfunction by inducing oxidative stress, inflammation, and disruption of the testicular immune microenvironment [2,7]. Lipopolysaccharide (LPS), a component of the cell wall of Gram-negative bacteria, can induce inflammatory responses in animals. Studies have shown that LPS can activate the TLR4/NF-κB signaling pathway to induce the release of pro-inflammatory factors and pyrin domain (PYD)-containing protein 3 (NLRP3) inflammasome activation in testicular tissue, leading to structural destruction of seminiferous tubules and spermatogenic cell apoptosis, ultimately causing male infertility [8]. Sertoli cells, as important somatic cells of the seminiferous tubules, not only provide nutritional support and structural scaffolding for germ cells, but also maintain the BTB by expressing tight junction proteins (such as Occludin and Claudin5) to protect the immune-privileged microenvironment in which germ cells develop. Studies have shown that LPS-induced inflammation reduces the expression of tight junction proteins in Sertoli cells, causing damage to the integrity of the BTB [9,10] and secretory dysfunction [11], thereby impairing spermatogenesis. The latest studies have shown that abnormal activation of NLRP3 inflammasome in Sertoli cells is significantly associated with cryptorchidism, while Vitamin D can inhibit NLRP3 activation and alleviate Sertoli cell damage [12], suggesting that targeting Sertoli cell inflammation may become a new strategy for the treatment of male infertility.

As a highly conserved degradation pathway within cells, autophagy maintains cellular homeostasis by removing damaged organelles and proteins. In the reproductive system, autophagy is involved in regulating BTB, maintaining Sertoli cell function, and promoting spermatogenesis [[13], [14], [15]]. In the LPS-induced cellular inflammation models, the expression of autophagy-related protein LC3-II was significantly upregulated, and autophagic activity was increased. This excessive activation of autophagy exacerbated cell damage [16]. Furthermore, bacteria-induced NLRP3 inflammasome activation can lead to sustained overactivation of autophagic flux [17]. Excessive autophagy disrupts BTB integrity by degrading tight junction proteins in Sertoli cells [18,19], inhibiting spermatogenesis and reducing sperm motility. It is worth noting that there is a bidirectional regulatory relationship between autophagy and inflammation [20]. Autophagy promotes NLRP3 inflammasome activation, exacerbating inflammatory injury [21]. Xu et al. [22] found that the formation of NLRP3 inflammasome depends on ULK1 activation. At the same time, studies have shown that autophagy induced by Resveratrol [23] and Berberine [24] can inhibit NLRP3 inflammasome activation and alleviate inflammatory injury. These may be related to the fact that autophagy promotes NLRP3 degradation and thus reduces NLRP3 inflammasome activation [25]. The crosstalk between autophagy and inflammation has been shown to be involved in the pathological process of infection, and autophagy has a dual role in NLRP3 activation and inflammatory response. However, the involvement of autophagy in the injury mechanism of LPS-induced Sertoli cell inflammation has yet to be examined.

Itaconate is produced by aconitate decarboxylase encoded by immune response gene 1 (IRG1). Recent studies have shown that itaconate and its derivative 4-octyl itaconate (4-OI) can exert anti-inflammatory effects [26]. Song et al. [27] showed that itaconate can enhance the anti-inflammatory ability of macrophages and inhibit atherogenesis. In addition, itaconate can inhibit the activation of STING signaling and reduce the production of inflammatory factors [28]. Itaconate also plays an important role in preventing oxidative stress. Itaconate improves liver antioxidant capacity by activating the nuclear factor erythroid 2-related factor 2 (Nrf2) signaling pathway [29]. Recent studies have found that itaconate promotes lysosome formation by alkylating transcription factor EB (TFEB), thereby enhancing the antibacterial ability of macrophages [30]. Furthermore, itaconate-mediated TFEB nuclear translocation promoted dysfunctional mitochondrial clearance by enhancing autophagic flux, thereby reducing AEC II cell apoptosis [31]. Tian et al. [32] proposed that itaconate can prevent renal fibrosis by reducing reactive oxygen species (ROS) and inhibiting autophagy activity. Wang et al. [33] also found that itaconate can inhibit autophagy and alleviate LPS-induced Glutathione peroxidase 4 (GPX4) autophagic degradation, thereby reducing HK-2 cell ferroptosis. These findings suggest that itaconate may exert protective effects synergistically through anti-inflammation, anti-oxidation, and autophagy regulation. Given the key role of Sertoli cell inflammation and abnormal autophagy in male infertility, and the low toxicity advantage of itaconate as an endogenous metabolite, the regulatory mechanism of itaconate on Sertoli cell autophagy and inflammatory injury urgently needs to be explored in depth. Because exogenous itaconate is difficult to transport across cell membranes into the cytoplasm, researchers synthesized and utilized the itaconate derivative 4-OI to explore its potential functions. 4-OI can enter cells and be hydrolyzed into itaconate, thereby exerting the biological effects of itaconate.

In this study, we demonstrated that 4-OI alleviated LPS-induced excessive autophagy in Sertoli cells and inhibited NLRP3 inflammasome activation by reducing ULK1 expression. Subsequently, 4-OI-inhibited autophagy promoted functional recovery of Sertoli cells following inflammation. Therefore, 4-OI may represent a promising therapeutic strategy for bacterial orchitis and inflammation-associated male infertility.

## 2 Materials and methods

### 2.1 Ethics statement

All experimental procedures were approved by the Institutional Animal Care and Use Committee of Zhejiang A&F University, China (ZAFUAC202474).

### 2.2 Cell treatment

TM4 Sertoli cells were purchased from Shanghai QiDa Biological Science and Technology Ltd (Shanghai, China) and cultured in DMEM/F12 (L310KJ, BasalMedia, Shanghai, China) supplemented with 10% fetal bovine serum (FBS, 900-108, GEMINI, CA, USA), 100 IU/mL penicillin, and 100 μg/mL streptomycin (C100C5, NCM Biotech, Suzhou, China) at 37 °C 5% CO_2_. TM4 Sertoli cells were characterized by immunofluorescence staining for the Sertoli cell markers SOX9, WT1 and ABP. The numbers of positive cells were quantified by counting cells exhibiting specific fluorescence signals. For LPS treatment, TM4 Sertoli cells were treated with 0.5, 1, 5, 10, and 20 μg/mL LPS [34] (L2880, Sigma, Hessen, Germany) for 24 h. To determine the effect of 4-OI, the concentrations of 4-OI (50, 100, and 150 μmol/L) were selected based on a previous study [35]. A stock solution of 4-OI (T4580, TargetMol, MA, USA) was prepared in dimethyl sulfoxide (DMSO) and diluted with culture medium to the indicated concentrations. The final concentration of DMSO in the culture medium was less than 0.1%. TM4 Sertoli cells were pretreated with 4-OI for 12 h, followed by exposure to 1 μg/mL LPS for 24 h. To examine the effect of autophagy on NLRP3, Sertoli cells were pretreated with the autophagy inhibitor chloroquine [36] (CQ, 50 μmol/L, T0194, TargetMol, MA, USA)or the autophagy activator rapamycin [36] (RAPA, 100 nmol/L, T1537, TargetMol, MA, USA) for 2 h and 1 h, respectively. Then, the cells were treated with 4-OI 12 h before LPS treatment. To determine the effect of NLRP3 on autophagy, Sertoli cells were pretreated with the NLRP3 inhibitor MCC950 [37] (10 μmol/L, T3701, TargetMol, MA, USA) for 4 h, then the cells were exposed to 1 μg/mL LPS for 24 h. To examine the effect of the transporter inhibitor N-(p-amylcinnamoyl) anthranilic acid (NAA, T5454, TargetMol, MA, USA), Sertoli cells were pretreated with 50 μmol/L NAA for 3 h [38], then treated with 50 μmol/L 4-OI. To interfere with ULK1 activity, Sertoli cells were pretreated with the ULK1 activator LYN-1604 [39] (1 μmol/L, T8808L, TargetMol, MA, USA) or the ULK1 inhibitor MRT68921 [40] (1 μmol/L, T6899, TargetMol, MA, USA) for 4 h before the cells were treated with 4-OI and LPS.

### 2.3 Western blot

Testis tissues and Sertoli cells were lysed with RIPA buffer (WB3100, NCM Biotech, Suzhou, China) supplemented with protease and phosphatase inhibitors (P002, NCM Biotech, Suzhou, China). After cell lysates were centrifuged at 12000 g for 15 min, total protein was collected and analyzed using a BCA kit (WB6501, NCM Biotech, Suzhou, China). 20 μg of protein was loaded into each lane and separated by a 10%-12% SDS-PAGE gel. Proteins were transferred to the PVDF membrane (IPVH00010, Millipore, Hessen, Germany) and blocked with 5% non-fat milk for 1 h. The membranes were incubated with primary antibodies (Table 1) overnight at 4 °C. β-actin (ACTB) served as an internal control. Then the membranes were incubated with HRP-conjugated goat anti-rabbit (1:14000, P8002, NCM Biotech, Suzhou, China) or anti-mouse IgG (1:14000, P8001, NCM Biotech, Suzhou, China) for 1 h at 37 °C. The bands were visualized by ECL (P10100, NCM Biotech, Suzhou, China) and analyzed using ImageJ software (Version 1.54p).

**Table 1:** Antibodies in the present study.

Antibodies	Dilution	Cat No.	Company
BAX	1:20000	ET1603-34	HUABIO, Hangzhou, China
BCL2	1:9000	12789-1-AP	Proteintech, Rosemont, IL, USA
Nrf2	1:1000	HA721432	HUABIO, Hangzhou, China
NLRP3	1:1500	ER1706-72	HUABIO, Hangzhou, China
NF-κB	1:10000	A19653	Abclonal, Wuhan, China
p–NF–κB	1:7000	AP1294	Abclonal, Wuhan, China
Beclin1	1:1500	WL02508	Wanleibio, Shenyang, China
p-Akt	1:3000	ab81283	Abcam, Cambridge, UK
Akt	1:4000	10176-2-AP	Proteintech, Rosemont, IL, USA
mTOR	1:20000	66888-1-Ig	Proteintech, Rosemont, IL, USA
p-mTOR	1:2000	ab109268	Abcam, Cambridge, UK
HO-1	1:3000	A1346	Abclonal, Wuhan, China
ATG5	1:7000	ET1611-38	HUABIO, Hangzhou, China
ULK1	1:1500	WL03067	Wanleibio, Shenyang, China
LC3	1:1500	ET1701-65	HUABIO, Hangzhou, China
p62	1:5000	HA721171	HUABIO, Hangzhou, China
GDNF	1:1000	ET1704-46	HUABIO, Hangzhou, China
Occludin	1:2000	WL01996	Wanleibio, Shenyang, China
ACTB	1:10000	R1207-1	HUABIO, Hangzhou, China

### 2.4 Cell viability assay

Cell viability was assessed using a Cell Counting Kit-8 (CCK-8, C0005, TargetMol, MA, USA) according to the manufacturer's instructions. TM4 Sertoli cells were seeded into 96-well plates at an appropriate density (5000 cells/well). After the indicated treatments, 10 μL of CCK-8 solution was added to each well and incubated at 37 °C for 3 h. The absorbance was measured at 450 nm using a microplate reader (Synergy H1, BioTek, VT, USA).

### 2.5 Lactate dehydrogenase (LDH) release assay

TM4 Sertoli cells were seeded in 96-well plates and exposed to LPS for 24 h with or without 4-OI pretreatment. LDH assay was performed using LDH Assay Kit (C0018S, Beyotime, Shanghai, China) according to the manufacturer's instructions. The absorbance of samples at 450 nm was measured using a microplate reader (Synergy H1, BioTek, VT, USA).

### 2.6 RNA preparation and quantitative real-time PCR (qRT-PCR)

Total RNA of TM4 Sertoli cells and testicular tissues was extracted by TRIzol reagent (Invitrogen, Carlsbad, CA) according to the manufacturer's protocols. cDNA was synthesized using 1 μg of RNA. qRT-PCR was performed by SYBR Green (G3326, Servicebio, Wuhan, China) using an Archimed R4 system (ROCGENE, Beijing, China). *β-actin* was used as an internal control. The data were analyzed using the 2-ΔΔCT method. The primer sequences are shown in Table 2.

**Table 2:** Primers in the present study.

Gene name	Forward primer	Reverse primer
*Caspase1*	TGAAAGACAAGCCCAAGGT	GGTGTTGAAGAGCAGAAAGCA
*NLRP3*	CTTGAAGAAGAGTGGATGGGTT	GCGTTCCTGTCCTTGATAGAGT
*Beclin1*	AGCCTCTGAAACTGGACACG	TAGCCTCTTCCTCCTGGGT
*Nrf2*	CTGGAAGTGTCAAACAGAACG	ACATTGGGATTCACGCATAG
*STRA8*	GGCAACCAACCCAGTGAT	AACTTATCCAGGCTTTCTTCC
*SOD2*	GGGAGCACGCTTACTACCTT	TTCTCCCAGTTGATTACATTCC
*NQO1*	AACTGGTTTACAGCATTGGC	TCTCCTCCCAGACGGTTTC
*HO-1*	AGGGTGACAGAAGAGGCTAAG	ACGCCATCTGTGAGGGACT
*ActivinA*	CCTGTCAGTAGTGGAGCGTG	AGACGGATGGTGACTTTGGT
*TGF-β1*	ACAACGCCATCTATGAGAAAAC	CCAAGGTAACGCCAGGAAT
*IGF1*	AGGCATTGTGGATGAGTGTTG	GAGCGGGCTGCTTTTGTA
*PGC-1α*	AGGTCCCCAGGCAGTAGAT	TCGTGCTCATAGGCTTCATAG
*FSHR*	TTCTTGTGCCAATCCTTTCC	GCACCTCATAACAGCCAAAC
*ABP*	CTGCTGTTGCTACTACTGATGC	GTTTGCTGATTTTGGTGAGG
*β-actin*	AGAGGGAAATCGTGCGTGAC	CGCTCGTTGCCAATAGTGAT

### 2.7 Immunofluorescence staining

TM4 Sertoli cells were seeded in 24-well plates and exposed to LPS for 24 h with or without 4-OI pretreatment. Then, Sertoli cells were fixed in 4% paraformaldehyde (P0099, Beyotime, Shanghai, China) and permeabilized with 0.1% Triton X-100 (P0096, Beyotime, Shanghai, China). For immunofluorescence staining of testicular tissues, paraffin-embedded sections were deparaffinized, rehydrated, and subjected to antigen retrieval after fixation in 4% paraformaldehyde. Thereafter, the sections were processed following the same staining protocol as described for TM4 Sertoli cells. After blocking with 10% goat serum (C0265, Beyotime, Shanghai, China), Sertoli cells and the sections were incubated with NF-κB (1:500, A19653, Abclonal, Wuhan, China), SOX9 (1:500, ET1611-56, HUABIO, Hangzhou, China), WT1 (1:800, GB150202, Servicebio, Wuhan, China), ABP (1:300, 18200-1-AP, Proteintech, Rosemont, IL, USA), PCNA (1:500, GB12010, Servicebio, Wuhan, China), c-Kit (1:500, GB153799, Servicebio, Wuhan, China), Laminin (1:500, GB15408, Servicebio, Wuhan, China), ZO-1 (1:300, GB15195, Servicebio, Wuhan, China), and LC3 (1:300, ET1701-65, HUABIO, Hangzhou, China) overnight at 4 °C. Secondary antibodies Goat anti-rabbit IgG (1:700, HA1122 and HA1121, HUABIO, Hangzhou, China) or Goat anti-mouse IgG (1:700, HA1125 and HA1126, HUABIO, Hangzhou, China) were used for signal detection. Cell nuclei were stained with DAPI. Sertoli cells were observed with a fluorescence microscope (CKX53SF, OLYMPUS, Tokyo, Japan). The fluorescence intensity was analyzed using ImageJ software (Version 1.54p).

### 2.8 Mitochondrial ROS measurement

Mitochondrial ROS were measured by MitoSOX Red (HY-D1055, MCE, NJ, USA). After the indicated treatments, cells were incubated with 5 μmol/L MitoSOX working solution for 30 min and washed with PBS. The images were observed and captured by a fluorescence microscope (CKX53SF, OLYMPUS, Tokyo, Japan). The fluorescence intensity was analyzed using ImageJ software (Version 1.54p).

### 2.9 Mitochondria staining

TM4 Sertoli cells were seeded in 24-well plates. Mitochondria were stained with 50 nmol/L Mito-Tracker Green (C1048, Beyotime, Shanghai, China) for 30 min at 37 °C. After staining, cells were washed with PBS and captured by a fluorescence microscope (CKX53SF, OLYMPUS, Tokyo, Japan). The fluorescence intensity was analyzed using ImageJ software (Version 1.54p).

### 2.10 PI fluorescent staining

TM4 Sertoli cells were pretreated with the ULK1 activator LYN-1604 or the ULK1 inhibitor MRT68921 for 4 h, followed by pretreatment with 50 μmol/L 4-OI for 12 h before exposure to 1 μg/mL LPS. The cells were incubated with PI working solution (C0080, Solarbio, Beijing, China) for 20 min at 37 °C. Cell nuclei were stained with Hoechst 33342. The images were captured by a fluorescence microscope (CKX53SF, OLYMPUS, Tokyo, Japan).

### 2.11 Protein half-life assay

ATG5, Beclin1, and ULK1 protein degradation was analyzed. TM4 Sertoli cells were seeded in 6-well plates. The cells were exposed to LPS for 24 h with or without 4-OI pretreatment. Then 150 μmol/L cycloheximide (CHX, T1225, TargetMol, MA, USA) was added to inhibit protein synthesis. After 0, 8, 16, and 24 h of CHX treatment, the cells were collected. Total proteins (20 μg) were separated by 10% SDS-PAGE gel. The membranes were incubated with anti-ATG5, anti-Beclin1, and anti-ULK1 antibodies (Table 1). The grayscale values of the bands were quantified and protein degradation curves were generated to analyze protein half-life.

### 2.12 Transmission electron microscopy (TEM)

After the indicated treatments, TM4 Sertoli cells were fixed with 2.5% glutaraldehyde (24 h, 4 °C). The samples were dehydrated with gradient alcohol and embedded in epoxy resin. Ultrathin sections (70 nm) were prepared and examined by TEM (HT7800, Hitachi, Tokyo, Japan) at 80 kV. The numbers of mitochondria and autophagosomes were counted.

### 2.13 Animal studies

Forty 10-week-old KM male mice were obtained from Charles River Laboratory Animal Technology Co. Ltd (Zhejiang, China). All mice were housed on a 12 h light/dark cycle with free access to food and water. Mice were randomly divided into four groups (n = 15): Ctrl, LPS, 4-OI, and LPS+4-OI groups. The mice in the 4-OI group were injected intraperitoneally with 50 mg/kg 4-OI (T4580, TargetMol, MA, USA) in 40% cyclodextrin/PBS, a dose selected according to a previous study [41]. The 40% cyclodextrin/PBS was the vehicle as control. After 12 h of 4-OI treatment, the mice were intraperitoneally injected with 10 mg/kg LPS to induce acute testicular inflammation [8]. After 24 h of LPS treatment, the mice were sacrificed by cervical dislocation and weighed immediately. Testicular tissues were collected to extract total RNA. Meanwhile, testicular and epididymal tissues were fixed in 4% paraformaldehyde and used for subsequent histological and immunofluorescence analyses. The cauda epididymides were collected for sperm quality evaluation. Briefly, epididymal sperm were released into pre-warmed PBS at 37 °C, and sperm concentration and motility were subsequently assessed using a computer-assisted sperm analysis (CASA) system (SongJingTianLun, Nanning, China).

### 2.14 Hematoxylin and eosin (HE) staining

Testicular and epididymal tissues were fixed in 4% paraformaldehyde, embedded in paraffin, and sectioned at 5 μm thickness. After deparaffinization and rehydration, the sections were stained with hematoxylin and eosin according to standard procedures. Histopathological changes were observed and photographed using a light microscope (CKX53SF, OLYMPUS, Tokyo, Japan).

### 2.15 TUNEL assay

Apoptotic cells in testicular tissues were detected using a TUNEL apoptosis detection kit (G1504, Servicebio, Wuhan, China) according to the manufacturer's instructions. Briefly, paraffin-embedded testicular sections were deparaffinized, rehydrated, and treated with proteinase K. The sections were then incubated with TUNEL reaction solution, followed by DAPI staining of nuclei. Images were captured using a fluorescence microscope (CKX53SF, OLYMPUS, Tokyo, Japan), the number of TUNEL-positive cells per seminiferous tubule was counted.

### 2.16 Sertoli cell isolation

Sertoli cells were isolated from the testes of each treatment group following a previously described protocol [42] with minor modifications. Briefly, testes were rinsed three times in PBS, and the tunica albuginea was carefully removed. The tissue was then minced into 1 mm^3^ fragments. The minced tissue was first digested with 2 mg/ml collagenase IV (BS165, Biosharp, Beijing, China) and 40 μg/ml DNase I (BS137, Biosharp, Beijing, China) at 37 °C for 20 min, followed by a second digestion using 0.25% trypsin-EDTA for 15 min. The cell suspension was passed through a 40 μm cell strainer (BD Falcon, CA, USA) to remove undigested debris. Isolated Sertoli cells were collected for protein extraction, and the expression levels of apoptosis-, autophagy-, and inflammation-related proteins were determined by Western blot analysis.

### 2.17 Statistical analysis

The data were shown as mean ± standard deviations (SD). Statistical analyses were performed by Student's t-tests or one-way analysis of variance using Graph Pad Prism 8.0. At least three independent experiments were performed. *p* < 0.05 were considered significant.

## 3 Results

### 3.1 4-OI alleviates LPS-induced NLRP3 activation and Sertoli cell inflammation

**Fig. 1:**
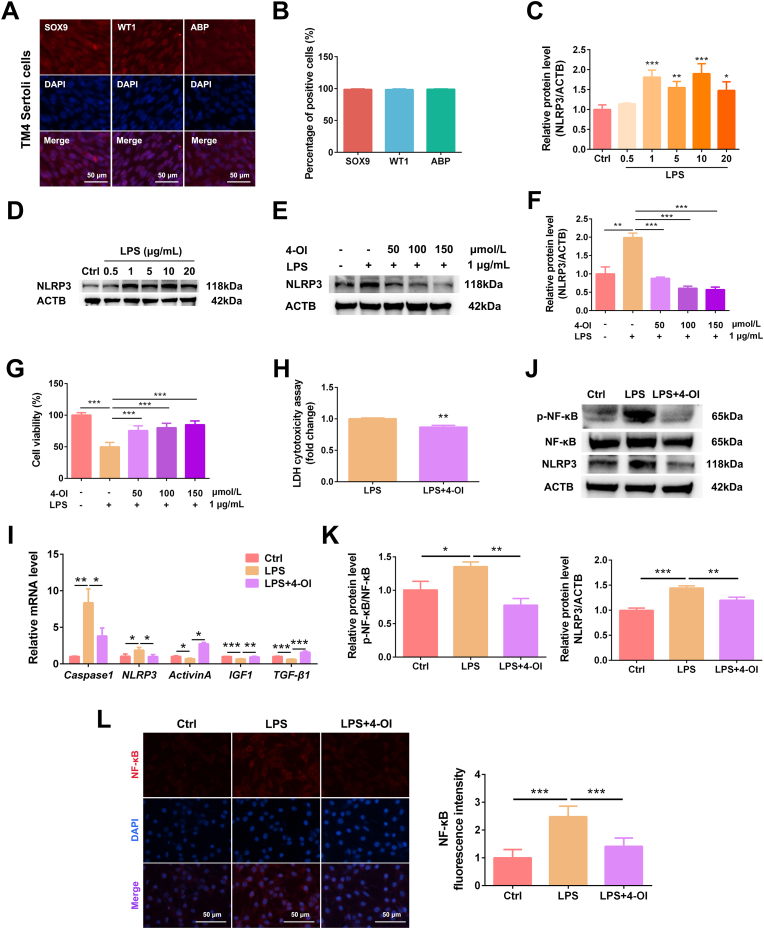
**4-OI alleviates LPS-induced NLRP3 activation and Sertoli cell inflammation.** A. Mouse TM4 Sertoli cells were identified by immunofluorescence staining using SOX9, WT1, and ABP as markers. Bar = 50 μm. B. The number of positive cells was counted (n = 5). C, D. TM4 Sertoli cells were treated with LPS (0, 0.5, 1, 5, 10, and 20 μg/mL). The expression levels of NLRP3 were analyzed by Western blot (n = 3). E, F. TM4 Sertoli cells were pretreated with 4-OI (50, 100, and 150 μmol/L), then incubated with 1 μg/mL LPS. NLRP3 expression levels were analyzed by Western blot (n = 3). G. After treatment with LPS or 4-OI, cell viability was analyzed by CCK-8 assay (n = 5). H. LDH cytotoxicity assay (n = 3). I. The mRNA expression levels of *NLRP3*, *Caspase1*, *TGF-β1*, *ActivinaA*, and *IGF1* were analyzed by qRT-PCR. J, K. TM4 Sertoli cells were pretreated with 50 μmol/L 4-OI, then exposed to 1 μg/mL LPS. The expression levels of p–NF–κB and NLRP3 were analyzed by Western blot (n = 3). L. Immunofluorescence staining of NF-κB in Ctrl, LPS, and LPS+4-OI groups (n = 5). Bar = 50 μm *p < 0.05, **p < 0.01, ***p < 0.001.

The mouse TM4 Sertoli cells were identified by immunofluorescence staining. The results showed that nearly all cells exhibited positive signals for SOX9, WT1, and ABP (Fig. 1A and B), indicating that these cells are Sertoli cells and are suitable for subsequent experiments. To explore the regulatory effect of 4-OI on Sertoli cells, TM4 Sertoli cells were treated with LPS to construct an inflammation model. The results showed that 1, 5, 10, and 20 μg/mL LPS significantly (*p* < 0.05) increased the expression level of NLRP3 (Fig. 1C and D). TM4 Sertoli cells were pretreated with 50, 100, or 150 μmol/L 4-OI, then cells were exposed to 1 μg/mL LPS to stimulate inflammatory response. The results showed that 4-OI pretreatment significantly (*p* < 0.001) decreased the expression level of NLRP3 (Fig. 1E and F) and increased (*p* < 0.001) the cell viability (Fig. 1G) in LPS-exposed Sertoli cells. And pretreatment with 50 μmol/L 4-OI significantly (*p* < 0.01) decreased the LDH levels compared with the LPS-treated group (Fig. 1H). Furthermore, we found that 4-OI treatment significantly reduced the mRNA expression levels of pro-inflammatory factors *NLRP3* and *Caspase1*, and increased the mRNA expression levels of anti-inflammatory factors *transforming growth factor-β* (*TGF-β1*), *ActivinA*, and *insulin-like growth factor 1* (*IGF1*), compared with the LPS-treated group (Fig. 1I). In addition, 4-OI administration significantly (*p* < 0.01) decreased the NF-κB phosphorylation level and NLRP3 protein level compared with the LPS-treated group (Fig. 1J and K). LPS exposure significantly increased (*p* < 0.001) NF-κB expression in the nuclei of Sertoli cells, while 4-OI treatment significantly inhibited (*p* < 0.001) NF-κB nuclear translocation (Fig. 1L). These results indicate that 4-OI can inhibit NLRP3 activation and alleviate LPS-induced inflammatory injury to Sertoli cells.

### 3.2 4-OI alleviates oxidative stress and promotes mitochondrial biogenesis

**Fig. 2:**
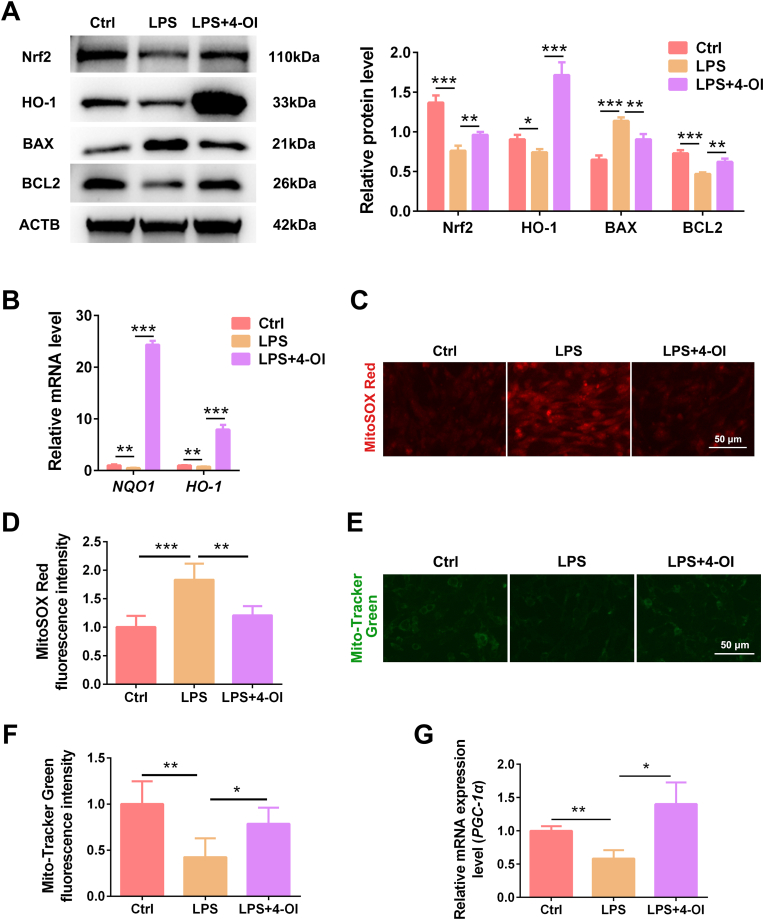
**4-OI alleviates oxidative stress and promotes mitochondrial biogenesis.** A. The expression levels of Nrf2, HO-1, BAX, and BCL2 were analyzed by Western blot (n = 3). B. The mRNA expression levels of *HO-1* and *NQO1* were analyzed by qRT-PCR. C. Mitochondrial ROS were analyzed using MitoSOX Red. Bar = 50 μm. D. The fluorescence intensity of MitoSOX Red (n = 5). E. Mitochondria were stained with Mito-Tracker Green. Bar = 50 μm. F. The fluorescence intensity of Mito-Tracker Green (n = 5). G. The mRNA expression level of *PGC-1α* were analyzed by qRT-PCR. *p < 0.05, **p < 0.01, ***p < 0.001.

LPS-induced inflammation causes oxidative stress, which exacerbates inflammatory injury. Therefore, we further analyzed the effect of 4-OI on oxidative stress in Sertoli cells. LPS exposure caused a decrease in the expression levels of Nrf2 and heme oxygenase 1 (HO-1) proteins (*p* < 0.05), while 4-OI treatment significantly (*p* < 0.01) increased the expression levels of Nrf2 and HO-1 (Fig. 2A). 4-OI treatment increased B-cell lymphoma-2 (BCL2) protein expression and decreased BCL2-associated X protein (BAX) protein expression compared with the LPS-treated TM4 Sertoli cells (Fig. 2A), indicating that 4-OI inhibited LPS-induced Sertoli cell apoptosis. qRT-PCR results also showed that 4-OI treatment significantly (*p* < 0.001) increased the mRNA expression levels of antioxidant genes *HO-1* and *NQO1* (Fig. 2B). Furthermore, 4-OI administration significantly reduced the mitochondrial ROS level compared with the LPS-treated group (Fig. 2C and D). Moreover, the results showed that 4-OI treatment resulted in increased Mito-Tracker Green fluorescence intensity (Fig. 2E and F) and *peroxisome proliferator-activated receptor-γ coactivator-1α* (*PGC-1α*) mRNA expression levels (Fig. 2G). Taken together, these data suggest that 4-OI alleviates LPS-induced oxidative stress and promotes mitochondrial biogenesis in Sertoli cells.

### 3.3 4-OI reduces LPS-activated autophagy to inhibit NLRP3 activation

**Fig. 3:**
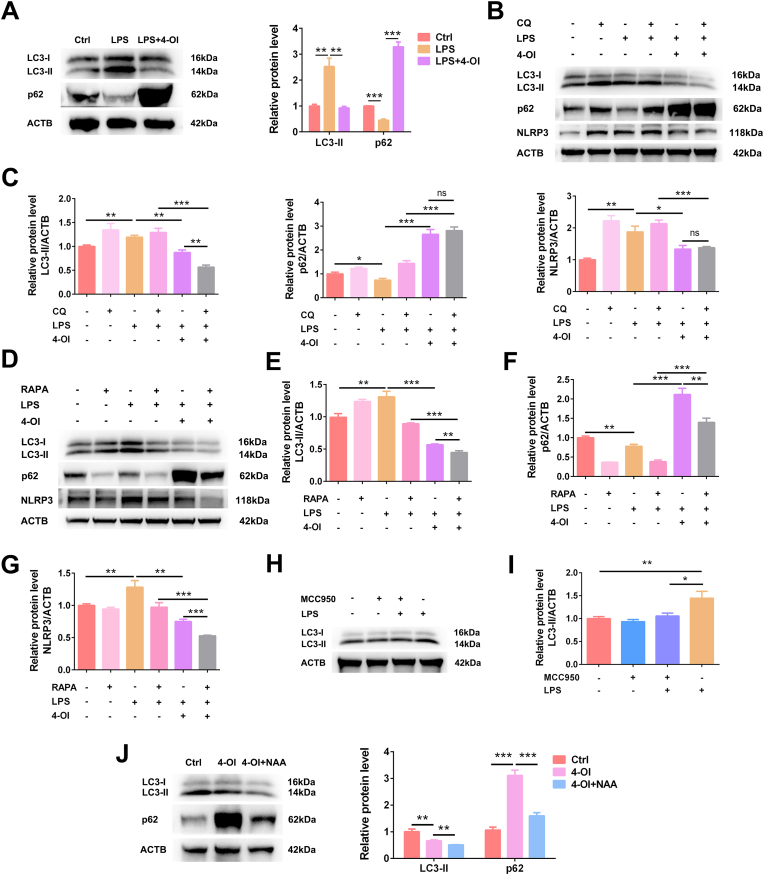
**4-OI reduces LPS-activated autophagy to inhibit NLRP3 activation.** A. The expression levels of LC3 and p62 were analyzed by Western blot (n = 3). B, C. TM4 Sertoli cells were pretreated with autophagy inhibitor CQ, then cells were incubated with 4-OI or LPS. The expression levels of LC3, p62, and NLRP3 were analyzed (n = 3). D-G. TM4 Sertoli cells were pretreated with autophagy activator RAPA, then cells were incubated with 4-OI or LPS. The expression levels of LC3, p62, and NLRP3 were analyzed (n = 3). H, I. Effect of NLRP3 inhibitor MCC950 on LC3 expression (n = 3). J. Effects of the transporter inhibitor NAA on LC3 and p62 expression (n = 3). ns, no significance. *p < 0.05, **p < 0.01, ***p < 0.001.

Autophagy plays an important role in maintaining cellular homeostasis under stress conditions. We found that LPS exposure significantly (*p* < 0.01) increased LC3-II expression and p62 degradation (Fig. 3A), indicating that LPS-induced inflammation leads to overactivation of autophagic flux in Sertoli cells. 4-OI administration reduced autophagic activity in LPS-treated Sertoli cells, as evidenced by decreased LC3-II expression levels and increased p62 protein accumulation (Fig. 3A). To determine the effect of autophagy on NLRP3 activation, Sertoli cells were treated with the autophagy inhibitor CQ or the autophagy activator RAPA. The results showed that CQ pretreatment inhibited autophagy activity (Fig. 3B and C) but had no significant effect on the reduced NLRP3 protein expression level in the LPS+4-OI group (*p* > 0.05) (Fig. 3B and C). Notably, RAPA-activated autophagy further exacerbated NLRP3 protein degradation (Fig. 3D–G), suggesting that interfering with autophagy may affect NLRP3 protein levels. Because autophagy modulation altered NLRP3 expression, we next investigated whether reciprocal regulation also exists between NLRP3 activation and autophagy. To further explore whether NLRP3 expression affects autophagy activity, Sertoli cells were pretreated with MCC950 to inhibit NLRP3 activity and then detect autophagy protein expression. We found that MCC950 pretreatment significantly reduced the expression level of LC3-II in LPS-treated Sertoli cells (Fig. 3H and I), indicating that NLRP3 inactivation can alleviate LPS-induced excessive autophagy in Sertoli cells. Having established the reciprocal relationship between autophagy and NLRP3 activation, we next investigated whether cellular uptake of 4-OI is required for its regulatory effect on autophagy. To further clarify whether 4-OI regulates autophagy activity by entering Sertoli cells through transporter SLC13A3, NAA was used to inhibit the activity of the Sertoli cell transporter SLC13A3. The results showed that NAA pretreatment resulted in decreased LC3-II expression and increased p62 protein levels compared with the 4-OI-treated group (Fig. 3J), indicating that the transporter inhibitor NAA interfered with the regulatory effect of 4-OI on autophagy in Sertoli cells. These results indicate that 4-OI can reduce LPS-activated autophagy and thus inhibit NLRP3 activation in Sertoli cells.

### 3.4 4-OI reduces autophagy activity by inhibiting autophagosome formation

**Fig. 4:**
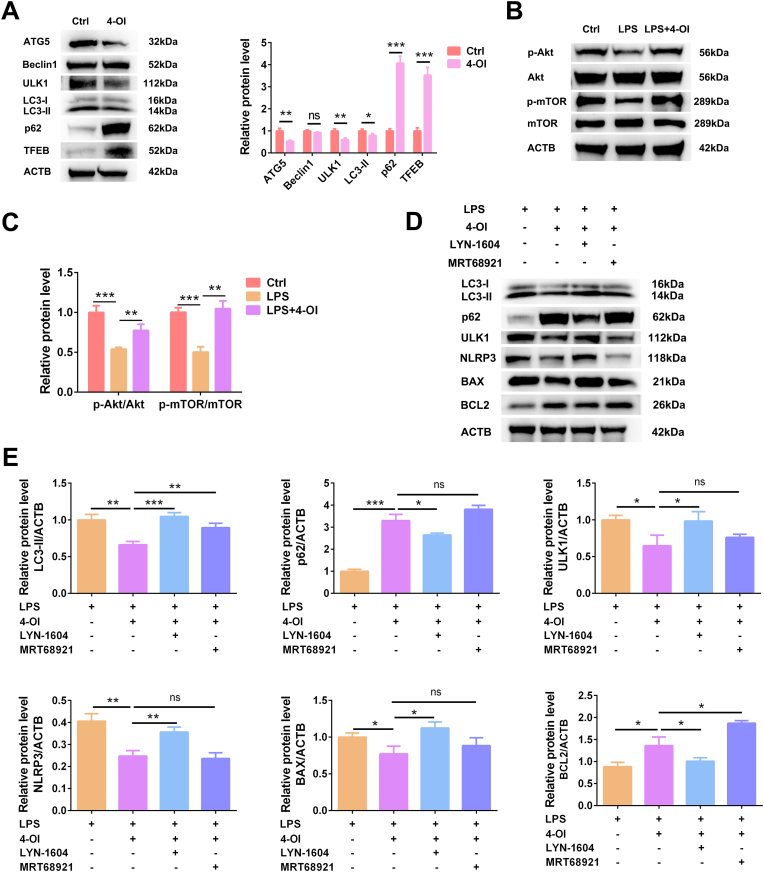
**4-OI reduces autophagy activity by inhibiting autophagosome formation.** A. TM4 Sertoli cells were treated with 50 μmol/L 4-OI. The expression levels of ATG5, Beclin1, ULK1, LC3, p62, and TFEB were analyzed by Western blot (n = 3). B, C. The phosphorylation levels of Akt and mTOR were analyzed by Western blot (n = 3). D, E. TM4 Sertoli cells were pretreated with ULK1 activator LYN-1604 or inhibitor MRT68921, then cells were incubated with 4-OI or LPS. LC3, p62, ULK1, NLRP3, BAX, and BCL2 protein levels were analyzed by Western blot (n = 3). ns, no significance. *p < 0.05, **p < 0.01, ***p < 0.001.

**Fig. 5:**
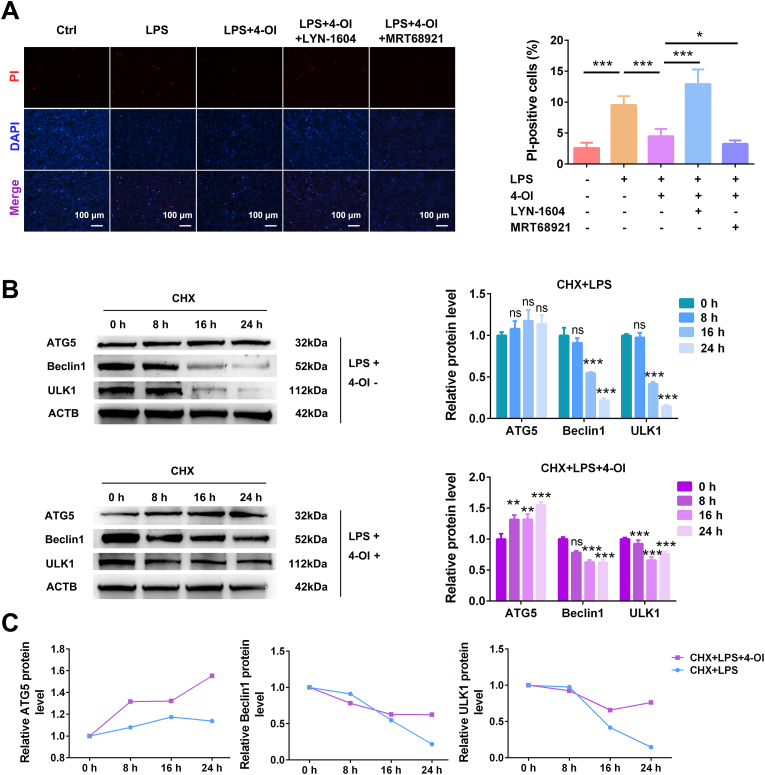
**4-OI inhibits Sertoli cell apoptosis and delays autophagic protein degradation.** A. Effects of the ULK1 activator LYN-1604 or inhibitor MRT68921 on LPS-induced apoptosis in Sertoli cells. Apoptotic Sertoli cells were stained with PI dye. PI-positive cells were counted (n = 6). Bar = 100 μm. B. TM4 Sertoli cells were pretreated with or without 4-OI, then cells were exposed to LPS. CHX were added to inhibit protein synthesis. After 0, 8, 16, and 24 h, the expression levels of ATG5, Beclin1, and ULK1 were analyzed by Western blot (n = 3). C. Half-life analysis of ATG5, Beclin1, and ULK1 proteins (n = 3). ns, no significance. *p < 0.05, **p < 0.01, ***p < 0.001.

To determine how 4-OI suppresses autophagy, we next examined the expression of key proteins involved in autophagosome initiation and autophagic flux. The results showed that 4-OI treatment significantly (*p* < 0.05) reduced the expression levels of ATG5, ULK1, and LC3-II, and increased the accumulation of p62 protein (Fig. 4A). The expression level of Beclin1 was not affected by 4-OI (*p* > 0.05, Fig. 4A), indicating that 4-OI inhibits the initiation of autophagy in Sertoli cells. mTOR, an important kinase regulating autophagy, can prevent ULK1 activation and thus inhibit autophagy initiation. Further results also showed that 4-OI treatment increased the phosphorylation levels of Akt and mTOR (*p* < 0.01) compared with the LPS-treated group (Fig. 4B and C). To verify whether ULK1 mediates the inhibitory effect of 4-OI on autophagy, we next pharmacologically manipulated ULK1 activity using the activator LYN-1604 and the inhibitor MRT68921. We found that treatment with the ULK1 activator partially abrogated the inhibitory effects of 4-OI on autophagy and inflammation, as demonstrated by increased ULK1 and NLRP3 expression levels and p62 protein degradation, compared with the LPS+4-OI group (Fig. 4D and E). Activation of ULK1 by LYN-1604 promoted BAX expression, decreased BCL2 expression (Fig. 4D and E), and increased the number of PI-positive cells (*p* < 0.001, Fig. 5A) compared with the LPS + 4-OI group. Treatment with the ULK1 inhibitor MRT68921 did not interfere with the inhibitory effects of 4-OI on autophagy and inflammation in Sertoli cells (Fig. 4D and E), suggesting that the anti-inflammatory and cytoprotective effects of 4-OI are associated with suppression of ULK1-mediated autophagy under conditions of inflammation-induced autophagic overactivation. In addition, we found that LPS-induced autophagy accelerated the degradation of Beclin1 and ULK1 proteins; whereas, 4-OI-treated Sertoli cells prolonged the half-lives of Beclin1 and ULK1 (Fig. 5B and C). Collectively, these findings suggest that 4-OI suppresses excessive autophagy primarily by inhibiting autophagosome formation.

### 3.5 4-OI-inhibited autophagy promotes functional recovery of Sertoli cells after inflammation

**Fig. 6:**
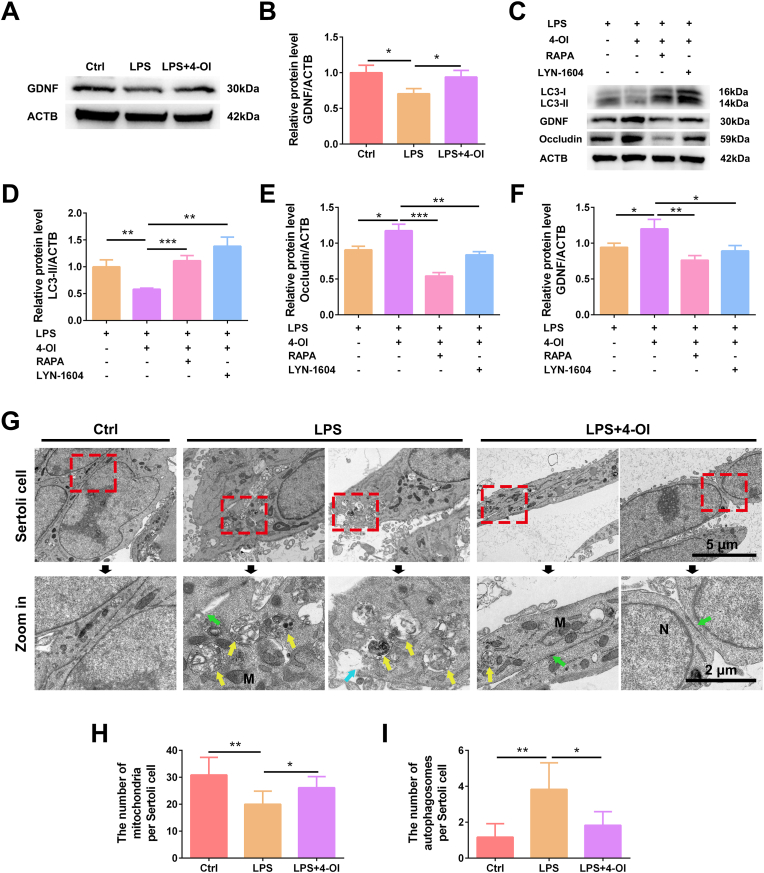
**4-OI-inhibited autophagy promotes functional recovery of Sertoli cells after inflammation.** A, B. The expression levels of GDNF were analyzed by Western blot (n = 3). C–F. TM4 Sertoli cells were pretreated with RAPA or LYN-1604, then cells were incubated with 4-OI or LPS. LC3, GDNF, and Occludin expression levels were analyzed by Western blot (n = 3). G. Sertoli cell ultrastructure and tight junctions were observed by TEM. Green arrows indicate tight junctions, yellow arrows indicate autophagosomes, and blue arrows indicate cell membrane rupture. M, mitochondria. N, nucleus. H, I. The number of mitochondria and autophagosomes in each Sertoli cell was counted (n = 6). *p < 0.05, **p < 0.01, ***p < 0.001.

Having established that 4-OI suppresses excessive autophagy, we next investigated whether this effect contributes to the functional recovery of Sertoli cells following inflammatory injury. The results showed that compared with the LPS-exposed group, 4-OI administration significantly (*p* < 0.05) increased glial-cell-line-derived neurotrophic factor (GDNF) protein expression levels (Fig. 6A and B). In addition, compared with the LPS+4-OI group, autophagy activated by RAPA and LYN-1604 offset the protective effect of 4-OI on Sertoli cell function and significantly reduced the expression levels of GDNF and Occludin (Fig. 6C–F). To further validate these findings at the ultrastructural level, Sertoli cells were examined by TEM. We found that LPS exposure led to rupture of the Sertoli cell membrane, damaged tight junction structure, decreased mitochondrial number (*p* < 0.01), and increased autophagosome number (*p* < 0.01) (Fig. 6G and H). However, 4-OI administration reduced the number of autophagosomes (*p* < 0.05), increased the number of mitochondria (*p* < 0.05), protected the integrity of cell membranes, and maintained the tight junction structure (Fig. 6G and H) These ultrastructural observations further support that 4-OI preserves Sertoli cell integrity and function by suppressing excessive autophagy during inflammatory injury.

### 3.6 4-OI alleviates LPS-induced inflammation and excessive autophagy in testicular tissues and isolated Sertoli cells

**Fig. 7:**
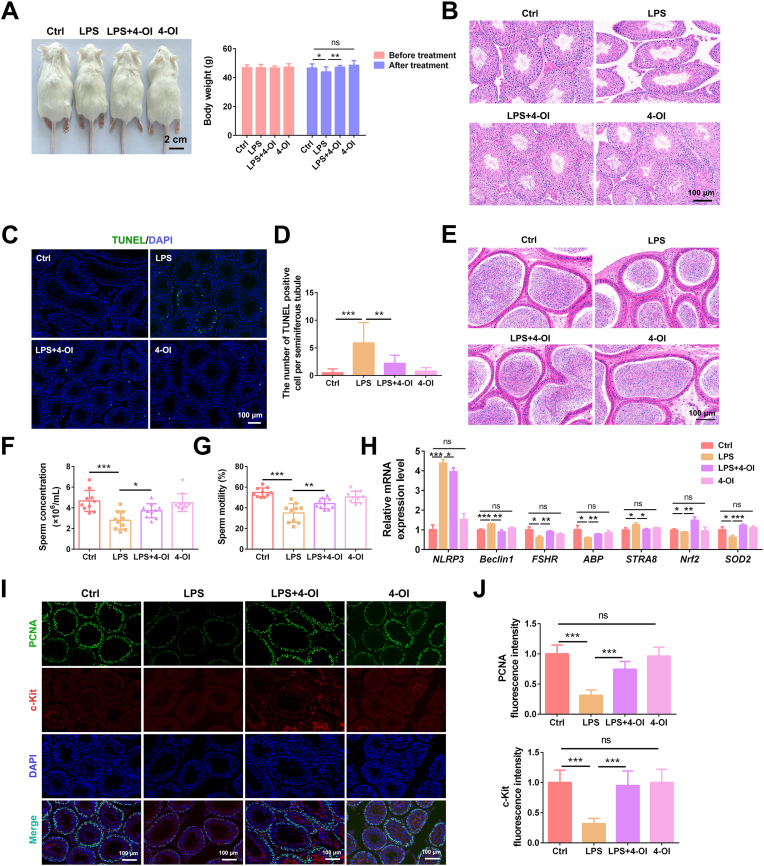
**4-OI alleviates LPS-induced inflammation and excessive autophagy in mouse testes.** A. Representative images of mice and mouse weight in different treatment groups (n = 10). B. HE staining in testes of different treatment groups. Bar = 100 μm. C. Representative images of TUNEL staining analysis. Bar = 100 μm. D. The number of TUNEL positive cell in per seminiferous tubule. E. HE staining in epididymis. Bar = 100 μm. F, G. Sperm concentration and motility in the epididymis of mice (n = 10). H. The mRNA expression levels of *NLRP3*, *Beclin1*, *Nrf2*, *SOD2*, *FSHR*, *ABP* and *STRA8* were analyzed by qRT-PCR in testis tissues of each treatment group (n = 5). I. Representative images of PCNA (green) and c-Kit (red) immunofluorescence staining in mouse testis. Bar = 100 μm. J. Quantitative analysis of PCNA and c-Kit immunofluorescence intensity (n = 5). ns, no significance. *p < 0.05, **p < 0.01, ***p < 0.001.

To validate the in vitro findings *in vivo*, mice were subjected to LPS-induced acute testicular inflammation, followed by evaluation of testicular pathology, spermatogenic function, and Sertoli cell response. We found that 4-OI pretreatment prevented (*p* < 0.01) the body weight loss in LPS-induced acute testicular inflammation (Fig. 7A). Histological examination of testicular sections by HE staining revealed that LPS challenge caused marked disruption of the seminiferous epithelium, characterized by epithelial disorganization, vacuolar degeneration, and impaired structural integrity of the seminiferous tubules. Notably, pretreatment with 4-OI markedly alleviated these pathological alterations and preserved the normal architecture of the seminiferous epithelium (Fig. 7B). Given the marked histopathological alterations, we next evaluated whether LPS-induced testicular injury was accompanied by increased germ cell apoptosis. To further evaluate cell apoptosis within the seminiferous tubules, TUNEL staining was performed. As shown in Fig. 7C and D, LPS exposure significantly increased the number of TUNEL-positive cells (*p* < 0.001), indicating enhanced apoptotic cell death in the testes. In contrast, 4-OI pretreatment markedly reduced (*p* < 0.01) the abundance of apoptotic cells induced by LPS (Fig. 7C and D). Because testicular injury is closely associated with impaired male fertility, we next assessed epididymal histology and sperm quality. HE staining of epididymal sections demonstrated that LPS treatment resulted in a pronounced reduction in sperm content within the epididymal lumen (Fig. 7E). Consistently, quantitative analysis showed that LPS significantly decreased (*p* < 0.001) sperm concentration and motility, whereas 4-OI pretreatment effectively improved (*p* < 0.05) both parameters (Fig. 7F and G). To further characterize the molecular alterations underlying these histological changes, we examined the expression of inflammation-, oxidative stress-, and Sertoli cell function-related genes in testicular tissues. qRT-PCR results showed that 4-OI pretreatment significantly decreased (*p* < 0.05) the mRNA expression levels of *NLRP3*, *Beclin1*, and *stimulated by retinoic acid gene 8* (*STRA8)*, and increased the mRNA expression levels of *Nrf2*, *SOD2*, *follicle-stimulating hormone receptor* (*FSHR*), and *androgen-binding protein* (*ABP*) in testicular tissues compared with the LPS exposure group (Fig. 7H).

**Fig. 8:**
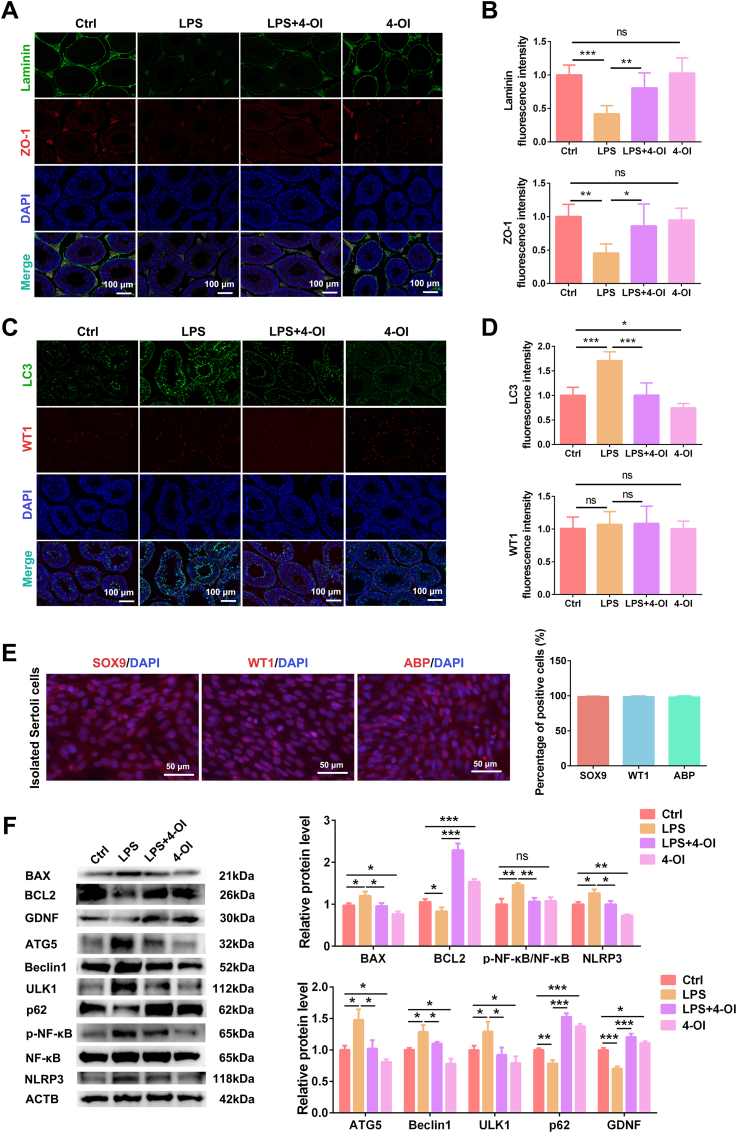
**4-OI protects Sertoli cell homeostasis and function in mouse testes.** A. Representative images of Laminin (green) and ZO-1 (red) immunofluorescence staining in mouse testis. Bar = 100 μm. B. Quantitative analysis of Laminin and ZO-1 immunofluorescence intensity (n = 5). C. Representative images of LC3 (green) and WT1 (red) immunofluorescence staining in mouse testis. Bar = 100 μm. D. Quantitative analysis of LC3 and WT1 immunofluorescence intensity (n = 5). E. Sertoli cells were isolated from the mouse testes and identified by immunofluorescence staining using SOX9, WT1, and ABP as markers. Bar = 50 μm. F. The expression levels of BAX, BCL2, GDNF, ATG5, Beclin1, ULK1, p62, p–NF–κB, and NLRP3 were analyzed by Western blot in isolated Sertoli cells from testes of each treatment group (n = 5). ns, no significance. *p < 0.05, **p < 0.01, ***p < 0.001.

To further assess testicular cellular activity and germ cell status, immunofluorescence staining was performed. The results demonstrated that LPS exposure markedly reduced (*p* < 0.001) the fluorescence intensity of PCNA and c-Kit in testicular tissues, whereas 4-OI pretreatment significantly restored (*p* < 0.001) their expression levels (Fig. 7I and J), indicating improved germ cell proliferation and differentiation. Compared with the LPS group, 4-OI significantly increased (*p* < 0.05) the fluorescence intensity of Laminin and tight junction protein ZO-1 (Fig. 8A and B), suggesting improved maintenance of the seminiferous basement membrane and BTB integrity. To further investigate Sertoli cell function and autophagy status, immunofluorescence staining of LC3 and WT1 was performed. The results revealed that LPS-induced activation of Sertoli cell autophagy was attenuated by 4-OI (Fig. 8C and D), as evidenced by a reduction in LC3 fluorescence intensity in WT1-positive cells (*p* < 0.001). To specifically determine whether the molecular changes observed in testicular tissues occurred in Sertoli cells, Sertoli cells were isolated from testes of different treatment groups. Immunofluorescence staining was performed to verify the purity of isolated Sertoli cells, and nearly all isolated cells were positive for Sertoli cell markers SOX9, WT1 and ABP (Fig. 8E). After confirming cell identity, the isolated Sertoli cells were subjected to Western blot analysis. Consistently, 4-OI administration significantly decreased (*p* < 0.05) the expression of pro-apoptotic (BAX), autophagy-related (ATG5, ULK1, Beclin1), and inflammatory (NLRP3) proteins, as well as the phosphorylation level of NF-κB (Fig. 8F). In contrast, the expression levels of anti-apoptotic BCL2, neurotrophic factor GDNF, and autophagy substrate p62 were markedly increased (*p* < 0.05) in Sertoli cells from the LPS+4-OI group (Fig. 8F). Collectively, these findings indicate that 4-OI alleviates LPS-induced testicular injury by suppressing excessive Sertoli cell autophagy and the associated inflammatory and apoptotic responses, thereby preserving Sertoli cell function, maintaining seminiferous epithelial integrity, and supporting normal spermatogenic activity.

## 4 Discussion

**Fig. 9:**
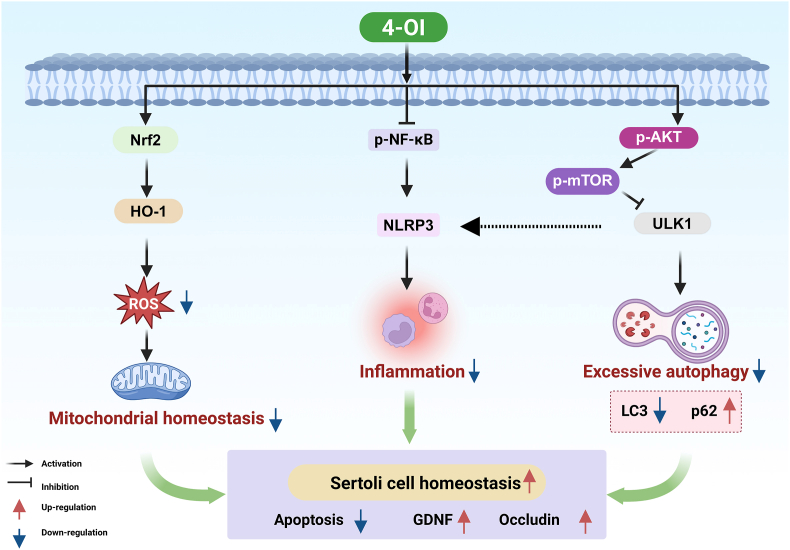
Proposed mechanism underlying the protective effects of 4-OI against LPS-induced Sertoli cell inflammation and dysfunction.

The maintenance of the testicular immune-privileged microenvironment depends on the normal function of Sertoli cells. Sertoli cells protect germ cell development by secreting growth factors and immunosuppressive factors and maintaining the integrity of the BTB. However, LPS stimulation can induce Sertoli cell inflammation, leading to BTB destruction, germ cell apoptosis, and male infertility [43]. Current therapeutic strategies for male reproductive inflammatory disorders mainly rely on antibiotics, hormonal interventions, or antioxidant supplementation. While these approaches may alleviate specific aspects of testicular injury, they generally do not simultaneously target inflammation, oxidative stress, and autophagy dysregulation. In contrast, 4-OI acts as an endogenous metabolite-derived compound capable of modulating multiple pathogenic pathways, suggesting a potential advantage in preserving Sertoli cell function and maintaining the spermatogenic microenvironment. In this study, in vitro and *in vivo* experiments confirmed that 4-OI significantly improved LPS-induced inflammation in testicular and Sertoli cells. Further mechanistic investigations revealed that 4-OI alleviated Sertoli cell inflammatory injury by suppressing excessive autophagy, thereby preserving Sertoli cell function and the spermatogenic microenvironment (Fig. 9).

As an endogenous metabolite of the tricarboxylic acid cycle, itaconate has been found in recent years to have broad-spectrum anti-inflammatory effects. Its mechanism involves inhibiting NF-κB, NLRP3 inflammasome, and regulating oxidative stress [26], but most of these studies have focused on macrophages. In the reproductive system, Sertoli cells serve as core regulators of the immune privilege microenvironment, but the mechanism of their inflammatory response and the role of itaconate have not yet been explored. Previous studies have shown that itaconate reduces the production of proinflammatory cytokines by inhibiting NF-κB nuclear translocation [44] and inhibits NLRP3 activation by directly modifying its cysteine residues [45]. To our knowledge, this study is the first to demonstrate that itaconate exerts potent anti-inflammatory effects in Sertoli cells, extending the well-established anti-inflammatory mechanism of itaconate to the male reproductive system. Following LPS stimulation, NF-κB phosphorylation and nuclear translocation were significantly increased, and NLRP3 expression was upregulated, while 4-OI treatment significantly inhibited these changes. Importantly, previous studies in Sertoli cells have primarily focused on the detrimental effects of inflammatory stimuli or the protective roles of antioxidants and hormonal regulators. In contrast, the present study identifies 4-OI as a novel metabolic regulator that simultaneously suppresses inflammation and excessive autophagy.

Autophagy plays a dual role in Sertoli cells. Studies have shown that autophagy protects Sertoli cells from oxidative stress under heat stress by clearing damaged mitochondria [46]. However, excessive autophagy induced by Zearalenone [19] or H_2_O_2_ [47] can lead to Sertoli cell dysfunction. In this study, LPS induced excessive activation of autophagy in Sertoli cells, which may be related to the acute inflammatory stimulation caused by LPS. TEM results showed that the number of autophagosomes decreased and the autophagic activity decreased after ITA treatment. It was further found that ITA reduced the expression of ULK1 and ATG5 and the degradation of p62 protein, indicating that ITA inhibited the initiation of autophagy in Sertoli cells and delayed degradation, which is consistent with previous research results [32,33]. These findings indicate that ITA affects autophagy by inhibiting autophagosome formation to reduce autophagic flux, thereby decreasing autophagic activity in Sertoli cells and alleviating LPS-induced inflammatory injury.

The relationship between autophagy and inflammation has been a research hotspot in recent years. Moderate autophagy can inhibit inflammation by clearing NLRP3 inflammasomes [48]. In this study, excessive autophagy promoted the activation of NLRP3, and its mechanism may be related to the release of ROS by autophagosome rupture. Our results showed that LPS exposure significantly increased mitochondrial ROS levels, while 4-OI treatment reduced ROS generation by inhibiting autophagy. This is consistent with the results of Wu et al. [49], indicating that ROS can stimulate NLRP3 activation and promote an inflammatory response. Previous studies have focused more on the direct inhibitory effect of 4-OI on inflammatory signals, while ignoring the regulatory role of autophagy in inflammation. In this study, LPS-induced inflammation in Sertoli cells was accompanied by excessive autophagy (increased LC3-II and p62 degradation), while 4-OI reduced autophagosome formation by inhibiting ULK1 expression, thereby alleviating inflammation, indicating that the anti-inflammatory effect of ITA in Sertoli cells depends on the synergistic effect of autophagy inhibition. This result is consistent with the finding reported by Li et al. that excessive autophagy promotes inflammatory injury [50]*.* However, our findings differ from those of Liu et al. [31], in that itaconate reduces AEC II cell apoptosis by enhancing autophagy to clear dysfunctional mitochondria. The reason for this difference may be due to exogenous stimuli and the degree of cellular stress. In this study, LPS-induced inflammation is acute and severe, during which autophagy is overactivated, exceeding the clearance capacity of Sertoli cells. In contrast, hyperoxia-induced injury in AEC II cells is chronic and mild, in which case insufficient autophagy is the primary issue, and 4-OI-promoting autophagy may play a protective role.

ULK1 is a core component of the autophagy initiation complex, and its activity is regulated by the Akt/mTOR pathway. This study found that ULK1 is a key target of 4-OI in regulating autophagy. 4-OI inhibits ULK1 expression by activating the Akt/mTOR pathway, thereby inhibiting the initiation of autophagy in Sertoli cells. Our findings suggest that ULK1 is closely associated with the autophagy-inflammation axis in Sertoli cells and may function as an important upstream regulator linking autophagic activity to inflammatory responses. Inhibition of ULK1 expression by 4-OI resulted in decreased autophagy activity and blocked NLRP3 inflammasome activation, indicating that ULK1 may represent an important regulatory node linking autophagy and inflammation in Sertoli cells. The role of ULK1 in inflammation was discovered in asthma mice, where activation of the NLRP3 inflammasome is dependent on ULK1 expression [22]. To further validate the effect of ULK1 on Sertoli cell inflammation, we examined the effect of interfering with ULK1 activity on NLRP3 protein levels. We found that ULK1 expression levels were significantly increased in LPS-treated Sertoli cells, and both 4-OI and ULK1 inhibitors limited NLRP3 inflammasome activation. ULK1 activator reversed the inhibitory effect of 4-OI on NLRP3 expression. In addition, we found that ULK1 inhibition by 4-OI not only reduced autophagosome formation but also decreased NLRP3 inflammasome activation, suggesting that ULK1 may contribute to NLRP3 inflammasome activation, potentially through modulation of autophagic activity, consistent with previous studies [22]. Although ULK1 inhibition by 4-OI was accompanied by reduced NLRP3 activation, the present study does not establish a direct interaction between ULK1 and the NLRP3 inflammasome. Instead, our findings suggest that ULK1 may influence NLRP3 activation indirectly through modulation of autophagic activity. Further studies are required to determine whether ULK1 directly interacts with components of the NLRP3 inflammasome signaling complex. In addition, although the present study demonstrates that inhibition of excessive autophagy is closely associated with reduced inflammation, it cannot be excluded that other signaling pathways regulated by 4-OI, such as metabolic reprogramming, may also contribute to its protective effects.

This study found that the protective effect of 4-OI on Sertoli cells is not limited to suppressing inflammation but also maintains their core functions. 4-OI increased the expression of Occludin (a tight junction protein), and TEM confirmed the integrity of the tight junction structure. Similarly, ultrastructural preservation has been recognized as an important indicator of testicular protection in other models of reproductive toxicity, further supporting the importance of maintaining cellular integrity during testicular injury [51]. Studies have shown that inflammation-induced tight junction damage is a major cause of infertility in orchitis. In this study, 4-OI protected tight junction structures by both inhibiting autophagy (reducing the autophagic degradation of Occludin) and reducing inflammation-induced damage to tight junctions. In macrophages, 4-OI reduces inflammation by promoting autophagy to clear damaged organelles. While maintaining moderate autophagic activity in Sertoli cells is crucial for germ cell survival, excessive autophagy can disrupt their secretory function and tight junction structure. Moreover, the protective effect of 4-OI on Sertoli cells is more dependent on the regulation of autophagy, and this difference may be due to the specificity of cell function. Therefore, 4-OI inhibits excessive autophagy rather than promotes autophagy in Sertoli cells, which may be a key strategy for them to adapt to cellular functional requirements. Studies have shown that defective autophagy in Sertoli cells (*Atg5* knockout) leads to Sertoli cell dysfunction and exacerbates cadmium-induced germ cell apoptosis [52]. This study found that LPS-induced excessive autophagy also led to Sertoli cell dysfunction (damaged tight junctions and reduced GDNF production), while these indicators were restored after 4-OI inhibited autophagy. Furthermore, LPS-induced excessive autophagy accelerated the degradation of tight junction proteins and mitochondria in Sertoli cells, which may increase the susceptibility of cells to bacterial infection [53]. Although the present study employed an LPS-induced inflammation model, excessive autophagy and NLRP3 inflammasome activation are also involved in a variety of reproductive disorders, including bacterial orchitis, varicocele, obesity-associated infertility, environmental toxicant exposure, and aging-related testicular dysfunction. Therefore, modulation of excessive autophagy by 4-OI may represent a broader therapeutic strategy for inflammation-associated male reproductive disorders beyond acute bacterial orchitis.

In this study, 4-OI improved the function of Sertoli cells and alleviated inflammatory injury by inhibiting excessive autophagy, which is consistent with previous studies [50]. In summary, as an endogenous metabolite, 4-OI has the advantages of low toxicity and high bioavailability, and is expected to become a potential drug for the treatment of testicular inflammation and male infertility. From a translational perspective, preservation of Sertoli cell function represents an attractive therapeutic strategy because Sertoli cells are essential for maintaining the blood–testis barrier, supporting spermatogenesis, and regulating testicular immune privilege. By simultaneously targeting inflammation, oxidative stress, and excessive autophagy, 4-OI may provide a multifaceted approach for protecting the spermatogenic microenvironment and preserving male fertility. Nevertheless, several limitations should be acknowledged. First, both the in *vitro* and in *vivo* experiments employed a pretreatment protocol, which primarily evaluates the preventive rather than therapeutic effects of 4-OI. This experimental design was selected to investigate the molecular mechanisms underlying the protective effects of 4-OI during the initiation of inflammatory injury and is consistent with previous mechanistic studies of 4-OI. However, recent evidence has demonstrated that 4-OI also exerts anti-inflammatory effects when administered after LPS stimulation, suggesting its therapeutic potential in established inflammatory diseases. Therefore, future studies employing post-treatment paradigms at different intervention time points are warranted to determine whether 4-OI can reverse established testicular inflammation and further evaluate its translational potential for the treatment of orchitis and inflammation-associated male infertility. Second, the present study focused on an acute inflammatory model, and the long-term efficacy and safety of 4-OI remain to be determined. Future studies should evaluate the pharmacokinetics, reproductive toxicity, and long-term therapeutic effects of 4-OI in chronic models of male infertility. In addition, validation in human Sertoli cells and clinically relevant preclinical models will be necessary before clinical application can be considered. Taken together, these future studies will be important for translating the protective effects of 4-OI from experimental models to clinical applications in male reproductive disorders.

## 5 Conclusions

In conclusion, 4-OI effectively protects Sertoli cells and testes against LPS-induced inflammatory injury. Mechanistically, 4-OI suppresses NF-κB/NLRP3 signaling, activates the Nrf2 antioxidant pathway, alleviates oxidative stress, and promotes mitochondrial homeostasis. More importantly, 4-OI inhibits ULK1-associated excessive autophagy, thereby reducing inflammatory responses, preserving Sertoli cell function, and maintaining tight junction integrity. These protective effects ultimately contribute to the preservation of the spermatogenic microenvironment and improvement of sperm quality *in vivo*. Collectively, our findings identify excessive autophagy as a critical regulator of Sertoli cell inflammatory injury and reveal 4-OI as a promising therapeutic candidate for orchitis and inflammation-associated male infertility. Future studies should further evaluate the long-term efficacy, safety, and translational potential of 4-OI in clinically relevant models and human reproductive systems.

## Ethics statement

All experimental procedures were approved by the Institutional Animal Care and Use Committee of Zhejiang A&F University, China (ZAFUAC202474).

## Funding

This study was supported by the 10.13039/501100004731Zhejiang Provincial Natural Science Foundation of China (LQ24C170001), the 10.13039/501100001809National Natural Science Foundation of China (32402753), Young Elite Scientists Sponsorship Program of Zhejiang Provincial Association for Science and Technology (ZJSKXQT2025033) and Zhejiang A&F University Talent Initiative Project (2022LFR066).

## CRediT authorship contribution statement

**Yuan Li:** Data curation, Investigation, Writing – original draft, Writing – review & editing. **Xianglong Wang:** Data curation, Formal analysis, Methodology. **Haijuan Yang:** Formal analysis. **Feng Jiang:** Methodology. **Meihua Wang:** Formal analysis. **Yue Wang:** Methodology. **Dong Niu:** Writing – review & editing. **Huaming Xi:** Methodology, Resources, Supervision, Visualization, Writing – original draft, Writing – review & editing.

## Declaration of competing interest

The authors declare that they have no conflict of interest.

## Data Availability

The data that support the findings of this study are available from the corresponding author upon reasonable request.

## Abbreviations

4-OI, 4-octyl itaconate; BTB, Blood-testis barrier; LPS, Lipopolysaccharide; NLRP3, Pyrin domain (PYD)-containing protein 3; Nrf2, Nuclear factor erythroid 2-related factor 2; ULK1, UNC-51-like kinase 1; TFEB, Transcription factor EB; GPX4, Glutathione peroxidase 4; FBS, Fetal bovine serum; CQ, Chloroquine; RAPA, Rapamycin; NAA, N-(p-amylcinnamoyl) anthranilic acid; ACTB, β-actin; LDH, Lactate dehydrogenase; qRT-PCR, Quantitative real-time PCR; ROS, Reactive oxygen species; TEM, Transmission electron microscopy; IRG1, Immune response gene 1; PCNA, Proliferating cell nuclear antigen; TGF-β1, Transforming growth factor-β; IGF1, Insulin-like growth factor 1; HO-1, Heme oxygenase 1; BCL2, B-cell lymphoma-2; BAX, BCL-2-associated X protein; PGC-1α, Peroxisome proliferator-activated receptor-γ coactivator-1α; NAA, N-(p-amylcinnamoyl) anthranilic acid; ABP, Androgen-binding protein; FSHR, Follicle-stimulating hormone receptor; GDNF, Glial-cell-line-derived neurotrophic factor.
